# Supplementary material for: EnzML: multi-label prediction of enzyme classes using InterPro signatures
Source: BMC Bioinformatics. 2012 Apr 25;13:61. doi: 10.1186/1471-2105-13-61 (PMC3483700; doi:10.1186/1471-2105-13-61)
Supplement: Addtional file 5 — The Java code to format the data files, evaluate and predict. The file enzml_java_code.tar.gz contains the Java code used to format database data to ARFF and XML formats, to execute cross and train-test (jackknife) evaluations and to record evaluation results to database. More information is included in the readme.txt file and the Javadoc files. The code can be used with a MySQL database. To use a different database software, other JDBC drivers might be required. [file 1471-2105-13-61-S5.gz › java_code/enzml2011/doc/uk/ac/ed/inf/enzml/test/mulan/attributesfilter/AttributesFilteredDataSetGeneratorTest.html]

AttributesFilteredDataSetGeneratorTest


---


|  |  |  |
| --- | --- | --- |
| **Overview** \| **Package** \| **Class** \| **Use** \| **Tree** \| **Deprecated** \| **Index** \| **Help** | |  |
| **PREV CLASS**   **NEXT CLASS** | **FRAMES**    **NO FRAMES**     **All Classes** |  |
| SUMMARY: NESTED \| FIELD \| CONSTR \| METHOD | DETAIL: FIELD \| CONSTR \| METHOD |  |


---


## uk.ac.ed.inf.enzml.test.mulan.attributesfilter Class AttributesFilteredDataSetGeneratorTest

```
java.lang.Object
  uk.ac.ed.inf.enzml.test.mulan.attributesfilter.AttributesFilteredDataSetGeneratorTest
```

---

``` public class AttributesFilteredDataSetGeneratorTest extends java.lang.Object ```

---

**Constructor Summary**

`AttributesFilteredDataSetGeneratorTest()`


| **Method Summary** | |
| --- | --- |
| `void` | `getNewTestDataSetGenerator()` |
| `void` | `testCreateAttributeFactory()` |

**Methods inherited from class java.lang.Object**

`equals, getClass, hashCode, notify, notifyAll, toString, wait, wait, wait`

**Constructor Detail**

### AttributesFilteredDataSetGeneratorTest

```
public AttributesFilteredDataSetGeneratorTest()
```


**Method Detail**

### getNewTestDataSetGenerator

```
public void getNewTestDataSetGenerator()
```

---


### testCreateAttributeFactory

```
public void testCreateAttributeFactory()
```


---


|  |  |  |
| --- | --- | --- |
| **Overview** \| **Package** \| **Class** \| **Use** \| **Tree** \| **Deprecated** \| **Index** \| **Help** | |  |
| **PREV CLASS**   **NEXT CLASS** | **FRAMES**    **NO FRAMES**     **All Classes** |  |
| SUMMARY: NESTED \| FIELD \| CONSTR \| METHOD | DETAIL: FIELD \| CONSTR \| METHOD |  |


---
